# 

**Published:** 1917-12

**Authors:** 


					L
DECEMBER, 1917.
Vol. XXXV. No. 134.
I
B.r Trn,a : rn
'JAN 2919$$
THE
BRISTOL
MEDICOCHIRU RGICAL
PUBLISUE1) US'OKU TUB AUSPICES OF
THE BRISTOL MEDICO-CHIRURGICA L SOCIETY.
Editor: P. WATSON-WILLIAMS, M.D.
WITH WHOM ARE ASSOCIATED
J. MICHELL CLARKE, M.A., M.D., LL.D.,
J. LACY FIRTH, M.S., M.D., JAMES SWAIN, C.B., M.S., M.D.
J. A. NIXON, B.A., M.D., Assistant Editor.
Acting Editorial Secntary: W. A. SMITH, M A., M.B.
/
52&U55
BRISTOL: J. W. ARROWSMITH LTD.
London : Simpkin, Marshall, Hamilton, Kent & Co. Ltd.
Price One Shilling and Sixpence net.

				

